# Die Rolle der Angehörigen im Kontext des assistierten Suizids: Herausforderungen und Unterstützungsbedarf

**DOI:** 10.1007/s00103-026-04194-x

**Published:** 2026-02-09

**Authors:** Birgit Wagner, Laura Hofmann

**Affiliations:** Abteilung für Klinische Psychologie, Medical School Berlin, Rüdesheimer Straße 50, 14197 Berlin, Deutschland

**Keywords:** Depression, Hinterbliebene, Anhaltende Trauerstörung, Trauer, PTBS, Depression, Physician-assisted dying, Persistent grief disorder, Grief, PTSD

## Abstract

Die Entscheidung für einen assistierten Suizid kann für Angehörige mit erheblichen emotionalen Belastungen einhergehen. Trotz der vielfältigen psychosozialen Herausforderungen finden Angehörige in Forschung, Versorgung sowie in ethischen und gesellschaftlichen Diskursen zu diesem Thema bislang nur unzureichende Beachtung. Der vorliegende Artikel bietet einen Überblick über die Rolle der Angehörigen im Kontext des assistierten Suizids.

Bisherige Studien zeigen, dass Angehörige häufig mit moralischen Dilemmata, ambivalenten Emotionen sowie antizipatorischer Trauer konfrontiert sind. Darüber hinaus übernehmen sie oftmals organisatorische Aufgaben zur Unterstützung der sterbewilligen Person und erleben nicht selten Stigmatisierung sowie daraus resultierende soziale Isolation. Zudem deuten bisherige Studienergebnisse darauf hin, dass Angehörige ein erhöhtes Risiko für die Entwicklung psychischer Erkrankungen aufweisen können.

Gleichzeitig können bestimmte Merkmale des assistierten Suizids entlastend auf Angehörige wirken und die Verarbeitung des Verlusts positiv beeinflussen. Hierzu zählen unter anderem die Möglichkeit zur Abschiednahme, offene Kommunikation sowie die Einbindung in den Prozess des assistierten Suizids. Diese Befunde unterstreichen die Notwendigkeit spezifischer psychosozialer Unterstützungsangebote für Angehörige sowie deren frühzeitige Einbindung in relevante Prozesse.

## Abstract (en)

The decision to pursue physician-assisted dying can place a significant emotional burden on relatives. Despite the considerable psychosocial stress they often face, these individuals have received limited attention in research, clinical practice and ethical or societal debates surrounding this topic. This article provides an overview of the role of relatives in the context of physician-assisted dying.

Previous studies indicate that relatives are frequently confronted with moral dilemmas, ambivalent emotions and anticipatory grief. In addition, they often take on organisational responsibilities in support of the person wishing to die and may experience stigma and subsequent social isolation. Research also suggests that relatives and close friends are at increased risk for mental health challenges.

At the same time, certain aspects of the process, such as the opportunity to say goodbye, open communication and the active involvement of relatives, can have a positive effect and support a more adaptive grieving process. These findings highlight the importance of psychosocial support for relatives as well as the necessity of involving them at an early stage throughout the entire process.

## Hintergrund

Seit der Legalisierung des assistierten Suizids stehen ethische und moralische Fragestellungen, die Ausgestaltung gesetzlicher Regelungen, die öffentliche Akzeptanz sowie mögliche Zugangsbeschränkungen für bestimmte Personengruppen im Mittelpunkt der gesellschaftlichen Debatte. Dabei richtet sich die Aufmerksamkeit überwiegend auf die sterbewillige Person, während die Perspektiven und psychischen Belastungen der Angehörigen weitgehend unberücksichtigt bleiben. Obwohl der assistierte Suizid weiterhin ein seltenes Ereignis darstellt, ist ein kontinuierlicher Anstieg der Fallzahlen in den letzten Jahren zu verzeichnen, wodurch zunehmend auch Angehörige und nahestehende Personen betroffen sind [1].

Bisher gibt es trotz der zunehmenden gesellschaftlichen Relevanz des assistierten Suizids nur wenige Studien, die sich mit den psychosozialen Auswirkungen auf die Angehörigen und deren Rolle im Prozess des assistierten Suizids auseinandersetzen [2]. Die vorliegenden, überwiegend qualitativen Studien liefern wichtige Anhaltspunkte für das Verständnis der besonderen psychosozialen Belastungen von Angehörigen und bilden zugleich den Ausgangspunkt für die vorliegende Arbeit.

Der Verlust einer Person durch einen assistierten Suizid unterscheidet sich in zahlreichen Aspekten von Verlusten durch andere Todesumstände [3]. Ein Teil der Familienmitglieder wird häufig bereits frühzeitig in den Entscheidungs- und Beantragungsprozess involviert [2, 4]. Sie begleiten beispielsweise die sterbewillige Person zu Beratungsgesprächen bei der Sterbehilfeorganisation oder leisten Unterstützung beim Ausfüllen erforderlicher Unterlagen [5, 6]. Gleichzeitig verfügen nur wenige Angehörige über vertiefte Kenntnisse zum Ablauf eines assistierten Suizids sowie zu dessen rechtlichen Rahmenbedingungen und emotionalen Implikationen. Häufig berichten sie beispielsweise davon, dass auch die behandelnden Ärzt:innen unzureichend informiert sind (z. B. durch Aussagen wie „ein assistierter Suizid ist in Deutschland verboten“), was die eigene Verunsicherung zusätzlich verstärkt und die Sorge vor gesellschaftlicher Stigmatisierung erhöht. Ein assistierter Suizid stellt für Angehörige eine komplexe existenzielle Erfahrung dar, die mit vielfältigen emotionalen und moralischen Herausforderungen verbunden ist und das spätere Trauererleben sowie die Verarbeitung des Verlusts wesentlich beeinflussen kann [7].

Bisherige Studien liefern keine einheitlichen Ergebnisse, ob Angehörige im Kontext eines assistierten Suizids psychisch stärker belastet sind als nach anderen Verlustarten (z. B. Suizide, natürliche Todesumstände) oder ob bestimmte Merkmale des assistierten Suizids, wie zum Beispiel die Möglichkeit zur Verabschiedung oder die Vorbereitung auf den Verlust, entlastend wirken können und somit zu einer geringeren psychischen Belastung führen [2, 5, 8].

In ihrem systematischen Review geben Andriessen et al. [2] anhand von insgesamt 10 Studien einen Überblick über die Trauer von Angehörigen nach einem assistierten Suizid. Betroffene zeigten niedrigere oder ähnliche Ausprägungen von Trauer wie Hinterbliebene nach anderen Verlustarten. Komplikationen beim Sterbeprozess, fehlende Akzeptanz gegenüber der Entscheidung für den assistierten Suizid, Ängste sowie Stigma waren mit einer erschwerten Trauerverarbeitung verknüpft. Als entlastende Faktoren werden die Möglichkeit, sich zu verabschieden, eine offene Kommunikation über den Sterbewunsch und ein Gefühl der Kontrolle aufgeführt [9, 10].

Bislang gibt es keinen klaren rechtlichen oder strukturellen Rahmen, in dem die Rolle der Angehörigen beim assistierten Suizid geregelt ist. Die Einbindung des familiären oder sozialen Umfelds ist abhängig von verschiedenen Faktoren wie der Qualität der Beziehung, der Akzeptanz der Entscheidung für den assistierten Suizid oder auch der benötigten Unterstützung während des Prozesses. Vor diesem Hintergrund gibt der vorliegende Artikel einen Überblick über die Rolle von Angehörigen im Kontext von assistiertem Suizid. Der Fokus liegt hierbei auf spezifischen Belastungsfaktoren, den psychischen Folgen sowie dem daraus resultierenden Unterstützungsbedarf von betroffenen Angehörigen.

## Spezifische Belastungsfaktoren von Angehörigen

### Ambivalenz und Akzeptanz der Entscheidung

Die Entscheidung einer nahestehenden Person für einen assistierten Suizid kann Angehörige vor weitgreifende Herausforderungen und innere Konflikte stellen. Betroffene möchten zum einen den Wunsch der sterbewilligen Person akzeptieren und sie dabei unterstützen, andererseits steht diese Entscheidung im Konflikt mit ihren eigenen Wünschen und Vorstellungen [7]. Ein weiterer Einflussfaktor ist die oft erlebte Diskrepanz zwischen dem Wunsch nach assistiertem Suizid der sterbewilligen Person und der von den Angehörigen wahrgenommenen noch bestehenden Lebensqualität [11]. Wenn Angehörige die betroffene Person als stabil oder nicht terminal erkrankt wahrnehmen, wird der geplante assistierte Suizid oft als noch nicht notwendig erachtet und der Wunsch nach einem frühzeitigen Tod erscheint nur schwer nachvollziehbar.

In einer kürzlich publizierten Studie der Autorinnen [7] basierend auf den Ergebnissen einer spezialisierten Beratungsstelle für Angehörige im Kontext von assistiertem Suizid berichteten 30 % der Befragten von Schwierigkeiten, die Entscheidung der sterbewilligen Person zu akzeptieren. Die Akzeptanz scheint insbesondere dann höher zu sein, wenn eine terminale somatische Erkrankung vorliegt und Angehörige das Leid miterleben [12]. Auch wenn Angehörige die Entscheidung für einen assistierten Suizid grundsätzlich respektieren, besteht oftmals der Wunsch oder die Frage, ob nicht alternative Behandlungsmaßnahmen in Anspruch genommen werden können (z. B. Unterbringung in einer Pflegeeinrichtung, Inanspruchnahme von alternativen medizinischen Optionen). Im Rahmen der Beratungsstelle [7] gaben diesen Wunsch fast 2 Drittel der Angehörigen an. Diese Ambivalenz kann in Schuldgefühlen resultieren, vor allem wenn Angehörige das Gefühl haben, der sterbewilligen Person nicht genug Unterstützung angeboten zu haben [12].

Ambivalente Gefühle können jedoch auch mit organisatorischen Aufgaben verknüpft sein. Angehörige stehen vor der Entscheidung, in welchem Ausmaß sie die sterbewillige Person unterstützen und begleiten möchten und ob sie bei dem Sterbeprozess anwesend sein wollen [9].

Ambivalente Haltungen können zudem innerhalb der Familie auftreten. Während einige Familienmitglieder den assistierten Suizid unterstützen und den Wunsch der sterbewilligen Person mittragen, lehnen andere ihn entschieden ab oder stehen dem Vorhaben kritisch gegenüber [13].

### Moralisches Dilemma

Auch wenn Angehörige keine formale Mitentscheidungsbefugnis über die Durchführung eines assistierten Suizids haben, da die rechtliche Autonomie bei der sterbewilligen Person liegt, können sie dennoch einen intensiven inneren moralischen Entscheidungsprozess durchlaufen. Häufig trifft sie der Wunsch nach einem assistierten Suizid unvorbereitet, sodass sie zunächst eine eigene Haltung dazu entwickeln müssen. Dabei spielen persönliche Werte wie das Recht auf Selbstbestimmung, Verantwortungsgefühl oder religiöse Überzeugungen eine zentrale Rolle (z. B. „Kann ich den Wunsch akzeptieren?“ „Steht das in Einklang mit meinem Glauben?“).

Gleichzeitig können eigene Schuldgefühle, Ängste, fehlendes Wissen und Unsicherheiten über den Ablauf diese innere Auseinandersetzung zusätzlich beeinflussen. Die Angehörigen stehen mitunter vor der Herausforderung, die Spannung auszuhalten, die sich aus der Diskrepanz zwischen der Autonomie der sterbewilligen Person und den eigenen moralischen Überzeugungen ergibt.

Dieses moralische Dilemma zeigt sich insbesondere in der Zeit vor dem assistierten Suizid [9, 14]. In einer Studie von Gamondi et al. [9] berichteten Angehörige von einem Gefühl, eine soziale Norm zu verletzen und zum Tod der nahestehenden Person beizutragen. Solche moralischen Dilemmata können auch lange Zeit nach dem Versterben der nahestehenden Person durch assistierten Suizid bestehen und mitunter eine Rolle für die Trauerverarbeitung spielen [2, 15]. Angehörige fragen sich, inwiefern es ihre moralische Aufgabe sei, die sterbewillige Person von ihrem Wunsch abzuhalten [7].

### Organisatorische Verantwortung

Bei vorliegenden somatischen oder psychischen Erkrankungen übernehmen Angehörige häufig bereits im Vorfeld eine Betreuungsrolle und sind in die Pflege und Unterstützung der sterbewilligen Person eingebunden [2]. Angehörige sind entsprechend oftmals bereits durch diese Aufgaben psychisch belastet, bevor die Entscheidung für einen assistierten Suizid getroffen wird. Diese unterstützende Rolle führen sie häufig auch bei den Vorbereitungen zum assistierten Suizid fort [7, 10]. Dazu zählen unter anderem das Ausfüllen von Anträgen oder Formularen, die Informationsbeschaffung, das Begleiten zu Beratungsterminen sowie die Kommunikation mit Sterbehilfeorganisationen, Ärzt:innen oder Sterbebegleiter:innen.

Diese Verantwortung ist häufig mit erheblichem zeitlichen und emotionalen Aufwand verbunden und wird von Angehörigen oft als belastend erlebt, insbesondere, wenn sie diese Rolle trotz eigener ambivalenter oder ablehnender Haltung gegenüber dem assistierten Suizid übernehmen. Vor allem bei starken krankheitsbedingten Einschränkungen oder einem hohen Alter der sterbewilligen Person und damit verbundenen Schwierigkeiten, sich eigenständig um organisatorische Aufgaben zu kümmern, sehen sich Angehörige verpflichtet, die sterbewillige Person diesbezüglich zu unterstützen. In einer von den Autorinnen durchgeführten Studie [7] berichteten Angehörige, dass sie insbesondere die organisatorische Unterstützung der sterbewilligen Person als belastend und unangemessen erlebten, da diese im starken Kontrast zu ihrer eigenen Trauer und Ängsten stand.

Gleichzeitig haben Angehörige Befürchtungen hinsichtlich möglicher rechtlicher Konsequenzen aufgrund ihrer Unterstützung [6, 7]. Generell wissen sie wenig über die legalen Aspekte des assistierten Suizids und es existieren große Unsicherheiten aufgrund nachfolgender Untersuchungen durch die Polizei.

### Abschiednahme und Planung des Sterbetages

Eine Besonderheit des Versterbens durch den assistierten Suizid ist das Wissen um das genaue Todesdatum. Dies stellt Angehörige vor große emotionale Herausforderungen [2, 16]. Bisherige Studien zeigen, dass das Festlegen eines Sterbedatums von den Angehörigen in der Regel als große psychische Belastung erlebt und als surreal beschrieben wird [6, 10]. Häufig äußern sie den Wunsch, den Termin kurzfristig zu verschieben, oder bitten sich ein Vetorecht aus, wenn sie zur Durchführung des assistierten Suizid noch nicht bereit sind. In einer Studie von Beuthin et al. [10] beschrieben Angehörige das Vereinbaren eines konkreten Termins als befremdlich. In diesem Kontext stellen sich die Angehörigen die Frage, wie sie die letzten Tage vor dem assistierten Suizid verbringen oder wie sie die Abschiednahme gestalten sollen. Außerdem stehen sie vor der Entscheidung, ob sie beim Sterbeprozess anwesend sein möchten und an welchem Ort der assistierte Suizid stattfinden soll.

Sterbehelfer:innen deutscher Sterbehilfeorganisationen berichteten beispielsweise, dass die Planung erschwert werden kann, wenn Familienmitglieder die Durchführung im gemeinsamen Zuhause ablehnen [17]. Dennoch ist es vielen Angehörigen ein wichtiges Anliegen, dass der assistierte Suizid in einer ruhigen und würdevollen Atmosphäre stattfindet, um der sterbewilligen Person einen guten Tod zu ermöglichen [1, 13]. Dies bedeutet für die Angehörigen häufig, ihre eigenen Gefühle von Trauer und Verzweiflung zunächst zurückzustellen und sich diese am Tag des assistierten Suizids gegenüber der sterbewilligen Person nicht anmerken zu lassen. Allerdings kann ein festgelegter Termin auch dabei helfen, sich bewusst in den Wochen davor von der nahestehenden Person zu verabschieden, mögliche Konflikte zu lösen oder intensiv Zeit miteinander zu verbringen [2]. Auch das bewusste Verbringen gemeinsamer Zeit wird von manchen Angehörigen im Nachhinein als wertvoll wahrgenommen [6, 10].

### Der Sterbeprozess

Die Entscheidung darüber, welche Rolle sie beim Sterbeprozess einnehmen möchten, wird von Angehörigen als problematisch und ambivalent erlebt [6, 7]. Im Rahmen der Beratungsstelle [7] berichteten über 40 % der Teilnehmenden Schwierigkeiten mit der Entscheidung, ob sie beim assistierten Suizid anwesend sein möchten. Im Vordergrund stehen dabei vor allem Ängste, die angehörige Person sterben zu sehen, potenzielle Komplikationen beim Sterbeprozess oder einen qualvollen Tod miterleben zu müssen. Angehörige fühlen sich jedoch oftmals verpflichtet anwesend zu sein, da sie die nahestehende Person nicht alleine lassen möchten [7].

Angehörige fühlen sich häufig unvorbereitet und wissen wenig über den Sterbeprozess und den konkreten Ablauf beim assistierten Suizid (z. B. „Was passiert genau, wenn das Medikament eingenommen wird?“). In einer Studie von Beuthin et al. [10] gaben Angehörige an, von dem schnellen Eintritt des Todes überrascht worden zu sein und sich in der Situation unvorbereitet gefühlt zu haben.

Zudem können während des Sterbeprozesses, abhängig von dem verabreichten Medikament, Komplikationen auftreten [18]. Eine Studie von Gleich et al. [18] zeigte, dass es bei bestimmten Medikamenten im Rahmen eines assistierten Suizids zu einer Verzögerung von mehreren Stunden zwischen Einnahme und Todeseintritt kommen kann. Zudem wurden Komplikationen wie Erbrechen oder Aspiration beschrieben, Reaktionen, mit denen die Angehörigen häufig nicht gerechnet hatten. Solche unerwarteten Ereignisse stellen für anwesende Angehörige eine erhebliche psychische Belastung dar, insbesondere wenn keine ärztliche Begleitung vor Ort ist und sie sich mit dem Ablauf des Sterbeprozesses überfordert fühlen.

### Polizeiliche Ermittlung

Da ein assistierter Suizid in Deutschland als „unnatürlicher Tod“ gilt, folgt unmittelbar eine medizinisch-juristische Untersuchung durch die Behörden. Die polizeilichen Abläufe nach einem assistierten Suizid werden von vielen Angehörigen als belastend beschrieben. Ein zentrales Problem besteht immer wieder in der mangelnden Kenntnis der rechtlichen Grundlagen zum assistierten Suizid bei den dazu gerufenen Polizist:innen [19]. Dies führt nicht selten zu Unsicherheiten und unangemessenen Reaktionen gegenüber den Angehörigen, etwa wenn Polizeibeamt:innen Unkenntnis über das Urteil des Bundesverfassungsgerichts aufweisen oder mit kugelsicheren Westen und Waffen in die Wohnung der verstorbenen Person eintreten [17].

Auch in der Schweiz berichteten Angehörige von belastenden Erfahrungen im Zusammenhang mit der Einschaltung von Polizei und Staatsanwaltschaft sowie der medizinischen Untersuchung des Leichnams, insbesondere in der sensiblen Phase der Abschiednahme [1]. Einige Betroffene äußerten das Gefühl, sich rechtfertigen zu müssen oder gar als Mitverantwortliche für den Tod angesehen zu werden. Eine Schweizer Studie von Wagner et al. [19] zeigte, dass problematische forensische Abläufe durch die Polizei, Staatsanwaltschaft und Gerichtsmedizin mit Symptomen einer posttraumatischen Belastungsstörung (PTBS), Depression und Trauer in Zusammenhang standen. Die Studienlage verdeutlicht, dass die Art und Weise, wie behördliche Untersuchungen durchgeführt werden, einen erheblichen Einfluss auf das psychische Wohlbefinden der Angehörigen haben kann.

### Geheimhaltung, soziale Isolation und Stigmatisierung

Während einige Studien keine Unterschiede in der erlebten Stigmatisierung von Angehörigen im Kontext des assistierten Suizids feststellen konnten [3], weisen andere auf ein erhöhtes Risiko stigmatisierender Erfahrungen hin [4, 20]. Oftmals sprechen Angehörige nicht offen über den assistierten Suizid in ihrem sozialen Umfeld oder verschweigen die Art des Versterbens aus Angst vor negativen Reaktionen oder Ablehnung auch innerhalb der Familie [9]. In einigen Fällen werden sie bereits im Vorfeld von der sterbewilligen Person gebeten, mit Dritten nicht über den assistierten Suizid zu sprechen [20].

Stigmatisierung und soziale Isolation können wiederrum zu Problemen mit der Trauerverarbeitung führen und einen Einfluss auf die Entstehung psychopathologischer Symptome haben [21]. Wagner et al. [21] fanden in ihrer Studie einen Zusammenhang zwischen fehlender sozialer Wertschätzung von Hinterbliebenen nach assistiertem Suizid und Symptomen einer PTBS und anhaltender Trauer.

Einen Überblick über die möglichen Belastungserfahrungen von Angehörigen bei assistiertem Suizid bietet die Infobox 1.

## Psychische Folgen für Angehörige

Es existieren bisher nur wenige quantitative Studien, die psychische Erkrankungen als Folge des assistierten Suizids erfassten. In der Studie der Autorinnen [7] wurden sowohl Angehörige vor als auch nach dem assistierten Suizid eingeschlossen. Fast die Hälfte der Teilnehmenden zeigte eine leichte und fast ein Drittel eine mittelgradige depressive Symptomatik. Insgesamt gaben 78 % Trauer als zentrales Thema an, während fast 50 % von Ängsten hinsichtlich des assistierten Suizids berichteten. In einer Studie von Ganzini et al. [3] mit insgesamt 95 Angehörigen nach assistiertem Suizid in den USA erfüllten 11 % die Kriterien für eine depressive Störung, über ein Drittel erhielt psychosoziale Unterstützung und bei 2 % lag eine anhaltende Trauerstörung vor. 38 % der befragten Teilnehmenden nahmen psychiatrische oder psychotherapeutische Unterstützung nach dem assistierten Suizid in Anspruch.

In einer Studie aus den Niederlanden [22] wurden Hinterbliebene (*n* = 189) von Patient:innen, die mittels Euthanasie verstarben, mit Personen (*n* = 316) aus dem Umfeld von Tumorpatient:innen, die eines natürlichen Todes gestorben waren, verglichen. Die Auswertung ergab, dass die Trauernden nach Euthanasie seltener unter intensiven Trauersymptomen und posttraumatischen Belastungsreaktionen litten als jene, deren Angehörige auf natürlichem Wege verstarben. In ihrer qualitativen Studie interviewten Snijdewind et al. [23] Personen, deren Partner:in aufgrund einer psychischen Erkrankung entweder durch Suizid (*n* = 15) oder durch assistierten Suizid (*n* = 12) gestorben war. Hinterbliebene nach assistiertem Suizid zeigten geringere Trauersymptome als Hinterbliebene nach Suizid.

Wagner et al. [24] untersuchten 85 Angehörige in der Schweiz, welche bei dem Sterbeprozess durch assistierten Suizid anwesend waren. Insgesamt erfüllten in dieser Studie 13 % die Kriterien einer PTBS und 6,5 % zeigten eine subklinische PTBS-Symptomatik, bei 16 % wurde eine depressive Symptomatik und bei 4,9 % eine anhaltende Trauerstörung diagnostiziert. Im Vergleich zur Allgemeinbevölkerung in der Schweiz wiesen Hinterbliebene in dieser Studie eine höhere Prävalenz der Depression und PTBS auf. Im Gegensatz dazu war die Prävalenz der anhaltenden Trauerstörung vergleichbar mit einer Schweizer Stichprobe der Allgemeinbevölkerung [25].

Zusammenfassend lässt sich festhalten, dass bislang nur wenige quantitative Studien zu den psychischen Auswirkungen eines assistierten Suizids vorliegen und sich die vorliegenden Studien durch Stichprobengröße, Art des begleitenden Sterbens (assistierter Suizid vs. Euthanasie) und Personengruppen wesentlich unterscheiden. In ihrer Übersichtsarbeit kamen Andriessen und Kolleg:innen [2] zu dem Ergebnis, dass der Trauerprozess nach einem assistierten Suizid oder Euthanasie im Vergleich zu anderen Todesumständen nicht negativ beeinflusst wird.

## Unterstützungsbedarf von Angehörigen

Obwohl Angehörige von sterbewilligen Personen häufig intensiv in den gesamten Prozess der Suizidassistenz eingebunden werden, existieren kaum spezifische Hilfsangebote für diese Personengruppe. Psychosoziale Unterstützungsangebote für Angehörige sind seitens der Sterbehilfeorganisationen bisher nicht etabliert. Im Folgenden werden Handlungsempfehlungen zur Unterstützung von Angehörigen im Kontext des assistierten Suizids vorgestellt, die auf Erfahrungen aus einer spezialisierten Beratungsstelle für diese Personengruppe basieren (siehe Infobox 2 und 3).

Zunächst ist es von zentraler Bedeutung, einen wertfreien und offenen Gesprächsrahmen für Angehörige zu schaffen. Viele Betroffene haben in der Vergangenheit entweder Stigmatisierung erfahren oder aus Angst vor Stigmatisierung nicht über den assistierten Suizid gesprochen [2, 20]. In einem geschützten Raum haben sie häufig erstmals die Möglichkeit, offen über den assistierten Suizid, die sterbewillige Person und ihre eigene Rolle zu sprechen. Bereits die offene Kommunikation und der Austausch mit einer nicht wertenden und mit dem Thema vertrauten Fachperson können für Betroffene entlastend wirken.

Häufig verfügen Angehörige nur über begrenztes Wissen hinsichtlich des Ablaufs, des Sterbeprozesses oder der rechtlichen Rahmenbedingungen des assistierten Suizids [6, 10]. Unzureichendes Wissen kann Ängste und Unsicherheiten verstärken und zu einer zusätzlichen psychischen Belastung führen. Eine neutrale und sachliche Informationsvermittlung über den Ablauf des assistierten Suizids kann Angehörige unterstützen. Auf dieser Basis können sie informierte Entscheidungen treffen. Darüber hinaus kann ein Gespräch mit dem zuständigen medizinischen Fachpersonal hilfreich sein, um spezifische Informationen (Wirkstoffgruppe, Darreichungsform) zu erhalten.

Vor dem assistierten Suizid berichtet die Mehrheit der Angehörigen von antizipatorischer Trauer, also einer Trauer, welche bereits vor dem Versterben einer Person auftritt [26]. In diesem Fall können psychoedukative Maßnahmen helfen, die Trauersymptome sowie andere auftretende Belastungsfaktoren zu normalisieren und einzuordnen.

Insbesondere die Planung des Abschieds sowie der Tage vor dem assistierten Suizid ist oftmals von Unsicherheiten geprägt [6, 7]. Angehörige berichten von Sorgen und Zweifeln, ob und wie diese Zeit besonders gestaltet werden sollte und wie sie sich von der nahestehenden Person verabschieden sollen. Hierbei kann es hilfreich sein, Erfahrungen von anderen Angehörigen einfließen zu lassen und herauszufinden, welche Aspekte für die betroffene Person bei der Abschiednahme besonders wichtig sind. Auf dieser Basis kann, wenn gewünscht, ein Plan für die Tage vor dem assistierten Suizid sowie für den Sterbetag entworfen werden. Das Gespräch zwischen sterbewilliger und angehöriger Person sollte gefördert werden, um die Bedürfnisse aller beteiligten Personen in die Gestaltung einbeziehen zu können.

## Fazit

Entscheidet sich eine Person für einen assistierten Suizid, kann dies für die Angehörigen mit zahlreichen emotionalen Belastungen einhergehen. Es zeigen sich in diesem Kontext zahlreiche psychosoziale Belastungsfaktoren, wie moralische Dilemmata, (antizipatorische) Trauer, Involviertheit in Entscheidungsprozesse, Akzeptanz und organisatorische Verantwortung. Hinzu kommt der Umgang mit dem Verlust, das Erleben des Sterbeprozesses sowie die anschließende Trauerverarbeitung. Während eine Reihe von Studien zeigte, dass Angehörige in Bezug auf ihre Trauerverarbeitung nicht stärker belastet sind als andere Hinterbliebenengruppen, gibt es dennoch zahlreiche Faktoren, die sich potenziell belastend auswirken können. Die öffentliche Wahrnehmung des assistierten Suizids sowie mögliche Stigmatisierungserfahrungen und daraus resultierende Isolation können den psychischen Stress verstärken. Viele Angehörige sind zudem in alle Phasen des assistieren Suizids eingebunden und stehen vor der Entscheidung, in welchem Ausmaß sie die sterbewillige Person unterstützen möchten. Zudem findet die Belastung von Angehörigen im Prozess des assistierten Suizids häufig keine Beachtung und es mangelt an etablierter psychosozialer Unterstützung, welche standardmäßig angeboten werden sollte.

Zusammenfassend kann gesagt werden, dass die Rolle der Angehörigen komplex und bisher unzureichend erforscht ist. Es fehlt an systematischen Längsschnittstudien, die die Belastung von Angehörigen sowie psychopathologische Symptome im Langzeitverlauf untersuchen. Vor diesem Hintergrund erscheint es empfehlenswert, die Perspektive der Angehörigen stärker in Forschung und Versorgung einzubinden.

### Infobox 1 Belastungserfahrungen von Angehörigen

- *Akzeptanz: *Schwierigkeiten, den Wunsch der sterbewilligen Person zu akzeptieren
- *Moralisches Dilemma:* Konflikt zwischen Respektieren des Wunsches der sterbewilligen Person und eigenen Werten und Wünschen
- *Organisatorische Verantwortung:* Unterstützung bei Formularen und Anträgen, Begleitung zu Terminen und Absprachen mit Sterbebegleiter:innen und Ärzt:innen
- *Angst vor rechtlichen Konsequenzen:* Unklarheiten und fehlendes Wissen hinsichtlich strafrechtlicher Risiken für Angehörige
- *Abschied:* Gestaltung der Tage vor dem assistierten Suizid und des Abschieds
- *Sterbeprozess:* Entscheidung über die Anwesenheit und den Ort der Durchführung des assistierten Suizids
- *Stigmatisierung und Isolation:* Geheimhaltung und fehlende Kommunikation, Angst vor oder erlebte Stigmatisierung

### Infobox 2 Unterstützungsbedarf von Angehörigen

- Offenen und wertfreien Raum schaffen
- Vermittlung von Informationen über die Abläufe eines assistierten Suizides
- Validierung der Trauer und ambivalenter Gefühle
- Psychoedukation über Trauerverläufe nach dem assistierten Suizid
- Entlastung bei Schuld- und Verantwortungsgefühlen
- Planung der Abschiednahme
- Anregung der Kommunikation mit sozialem Umfeld

### Infobox 3 Spezialisierte Beratungsstelle für Angehörige bei assistiertem Suizid

In der Spezialambulanz für Suizidprävention an der MSB Medical School Berlin wurde im Jahr 2022 von Prof. Dr. Birgit Wagner und Dr. Laura Hofmann eine spezialisierte Beratungsstelle für Angehörige im Kontext von assistiertem Suizid gegründet. Betroffene Personen können die Beratung vor und/oder nach dem assistierten Suizid in Anspruch nehmen. Die Beratung wird von psychologischen Psychotherapeutinnen durchgeführt. Das Beratungsangebot ist kostenlos und wird von einer wissenschaftlichen Studie begleitet.

Weitere Informationen und Anmeldung: https://www.medicalschool-berlin.de/forschung-lehrambulanz/spezialambulanz-suizidpraevention/.
